# The current standing of autologous haematopoietic stem cell transplantation for the treatment of multiple sclerosis

**DOI:** 10.1007/s00415-022-11063-5

**Published:** 2022-04-11

**Authors:** A. G. Willison, T. Ruck, G. Lenz, H. P. Hartung, S. G. Meuth

**Affiliations:** 1 Department of Neurology, Medical Faculty, University Hospital Düsseldorf, Heinrich Heine University, Düsseldorf, Germany; 2 Department of Medicine, University Hospital Münster, Albert-Schweitzer-Campus 1, Münster, Germany; 3 Department of Neurology, Medical University of Vienna, Vienna, Austria; 4 Brain and Mind Center, University of Sydney, Sydney, Australia; 5 Department of Neurology, Palacky University, Olomouc, Czech Republic

**Keywords:** Autologous haematopoietic stem cell transplant, Multiple sclerosis, Immune reconstitution

## Abstract

Autologous haematopoietic stem cell transplantation (aHSCT) is gaining traction as a valuable treatment option for patients affected by severe multiple sclerosis (MS), particularly the relapsing–remitting form. We describe the current literature in terms of clinical trials, observational and retrospective studies, as well as immune reconstitution following transplantation, with a focus on the conditioning regimens used for transplantation. The evidence base predominantly consists of non-randomised, uncontrolled clinical trials or data from retrospective or observational cohorts, i.e. very few randomised or controlled trials. Most often, intermediate-intensity conditioning regimens are used, with promising results from both myeloablative and lymphoablative strategies, as well as from regimens that are low and high intensity. Efficacy of transplantation, which is likely secondary to immune reconstitution and restored immune tolerance, is, therefore, not clearly dependent on the intensity of the conditioning regimen. However, the conditioning regimen may well influence the immune response to transplantation. Heterogeneity of conditioning regimens among studies hinders synthesis of the articles assessing post-aHSCT immune system changes. Factors associated with better outcomes were lower Kurtzke Expanded Disability Status Scale, relapsing–remitting MS, younger age, and shorter disease duration at baseline, which supports the guidance for patient selection proposed by the European Society for Blood and Marrow Transplantation. Interestingly, promising outcomes were described for patients with secondary progressive MS by some studies, which may be worth taking into account when considering treatment options for patients with active, progressive disease. Of note, a significant proportion of patients develop autoimmune disease following transplantation, with alemtuzumab-containing regimens associated with the highest incidence.

**Supplementary Information:**

The online version contains supplementary material available at 10.1007/s00415-022-11063-5.

## Introduction

Multiple sclerosis (MS) likely occurs against a background of genetic and environmental predisposition, leading to autoreactivity of peripheral immune cells, blood brain barrier (BBB) breakdown and central nervous system (CNS) infiltration [1–4]. Subsequent neuronal demyelination and neuroinflammation is facilitated by CNS-resident cells [5, 6]. Offering affected patients perhaps the most impressive series of therapeutic milestones over the past 28 years, MS research is a very encouraging example of bench-to-bedside success, at least for relapsing–remitting multiple sclerosis (RRMS). The progressive forms of MS—secondary progressive MS (SPMS) and primary progressive MS (PPMS)—have demonstrated a less encouraging response to novel treatments in clinical trials [7, 8]. Of note, PPMS may be considered “active” if relapses occur, which would have been referred to as progressive-relapsing MS (PRMS) prior to the publication of the 2013 International Advisory Committee on Clinical Trials of MS guidelines [9]. SPMS may also be termed “active” with reference to relapse occurrence [10]. These advancements in available treatments have allowed not only for a better understanding of disease pathogenesis, but also for no evidence of disease activity (NEDA) to become a realistic treatment target, particularly using immunomodulatory therapies.

Indeed, the approval of beta interferon (IFN-β) drugs in the 1990s as the first disease-modifying therapy (DMT) for RRMS accelerated MS research towards immunomodulatory approaches by demonstrating the efficacy of cytokine regulation and, as it was later elucidated, alteration of T cell subpopulations [11–14]. This confirmed that T cells did indeed facilitate relapses and could be therapeutically targeted. In this regard, key factors in the occurrence of relapse are thought to be autoreactivity of IL-17 expressing CD4^+^ T cells (i.e. T helper 17 cells (T_H_17)), CD4^+^ T helper 1 cells (Th1), CD8^+^ T cells and the insufficient function of regulatory T cells (T regs) [15–19], indicating that T cells are clearly important in MS pathogenesis. In addition, the recent advent of B cell-targeting monoclonal antibody therapies has established the critical role of, for example, pro-inflammatory CD20-expressing B cells in the cytokine cascade that characterises a relapse [20, 21] and daclizumab has more recently demonstrated a role for natural killer (NK) cells [22]. Not only does the infiltration of T cells and B cells contribute to relapse occurrence, but also that of macrophages and the activation of CNS-resident cells, including microglia and astrocytes, all of which contributing to pathogenic cytokine networks.

The early, now less efficacious, treatments, namely the IFN-β drugs and glatiramer acetate, still offer good outcomes as well as a favourable safety profile and, therefore, serve as an opener to “escalation therapy”, with severity of disease said to be proportional to the potency of treatment [23–26]. For those affected by particularly aggressive disease at a younger age, the hit hard and hit early approach, which may refer to “induction therapy” or “highly effective treatment early”, instead of escalation therapy is often considered, as efficacy is of the utmost importance for these patients. The key, here, is patient selection. Specifically, patients who will benefit the most from aggressive treatments in terms of disease outcome, i.e. successful immune reprogramming and long-term remission as well as tolerance of side effects are considered the optimal candidates. These highly efficacious treatments work by either sequestrating lymphocytes in lymph nodes, lymphocyte depletion (e.g. the so-called immune reconstitution therapies (IRTs)) or reducing immune cell trafficking into the CNS [27]. Following a short course of these immunomodulatory drugs, patients may then: be given either IFN-β or glatiramer acetate to maintain remission—this would be “induction therapy”; continue with the same immunomodulatory treatment; trial further highly efficacious treatments until symptoms are controlled. Compared to IFN-β and glatiramer acetate, the highly effective therapies have a significant side effect profile but are thought to confer a lower risk of developing SPMS [28, 29] and offer more favourable long-term clinical outcomes [30, 31].

Research being focussed on immunomodulation has, therefore, proven promising and there is good reason to more readily start highly effective immunomodulatory therapy [32]. MS is challenging to prognosticate; the natural history may occur insidiously over many years, of which a good number are often lived with minimal disability, but inflammation accumulates [33]. A patient’s disease burden may be underestimated by standard magnetic resonance imaging (MRI)—take the recently described smouldering lesions, for example, that are better visible with ultra-high field strength MRI but may be seen using 3 T or on histopathological examination [34]. Further, diagnosis is most often made between age 20–40, with these younger patients having fewer co-morbidities and thereby being more resilient to aggressive treatments. In this vein, for younger patients with early disease and lower Kurtzke Expanded Disability Status Scale (EDSS) score, increasing consideration in recent years has been given to autologous haematopoietic stem cell transplantation (aHSCT), a less well-accepted yet strikingly effective form of IRT that may allow for long-term, perhaps even life-long in some cases, cessation of aberrant immune system functioning [35, 36].

**Fig. 1:**
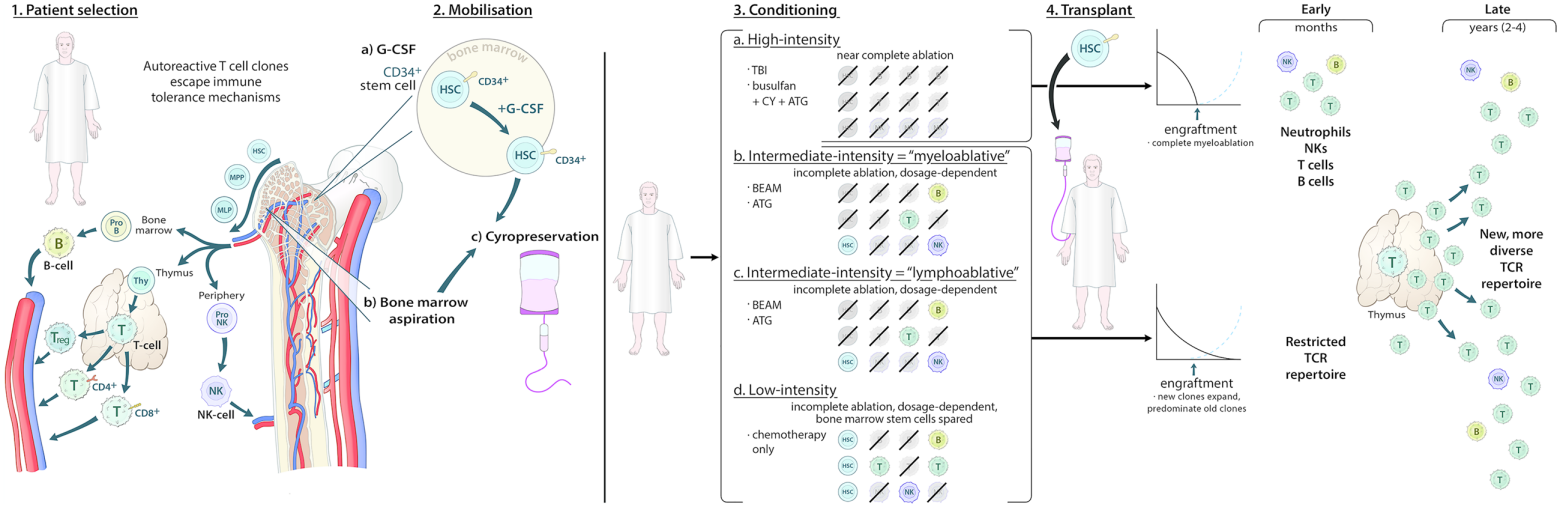
Autologous transplantation and immune reconstitution. The first stage is patient selection, where fitness and suitability for transplant are considered. In patients with MS, autoreactive T cell clones that have escaped immune tolerance mechanisms contribute to pathogenesis by effecting neuroinflammation, along with B cells and natural killer (NK) cells. These cells have common progenitors in the bone marrow, which are—at the earliest—haematopoietic stem cells (HSC), with later stages including multipotent progenitors (MPP) and multilymphoid progenitors (MLP). B cell maturation occurs primarily within the bone marrow. T cell maturation occurs within the thymus, with the bone marrow producing thymocytes that then undergo a complex maturation process within the thymus, producing regulatory T cells (Treg), CD4+ T cells and CD8+ T cells. Treg may also mature peripherally. NK cells begin their maturation process within the bone marrow, which is completed in the periphery. The transplant process then is initiated during mobilisation, where, most commonly in MS, HSC are either extracted peripherally (2a) following G-CSF (often with cyclophosphamide or rarely using cyclophosphamide alone) administration or, less commonly, from the bone marrow proper using bone marrow aspiration (2b). Cells are then cryopreserved (2c). The patient may then undergo conditioning, which may be of four intensities according to the European Society for Blood and Marrow Transplantation: high intensity, using total body irradiation (TBI) alone or in combination with other agents or busulfan with cyclophosphamide and anti-thymocyte globulin (ATG); intermediate-intensity “myeloablative” using BEAM + ATG; intermediate-intensity “lymphoablative” using cyclophosphamide + ATG; low-intensity using chemotherapy-only regimens, i.e. without ATG. For the high-intensity regimens, the conditioning should destroy all remaining immune cells and, therefore, at transplantation the patient has no immune cells being produced or in circulation, which is complete ablation. The other regimens will have varying degrees of immune cell destruction, with cells from the “old” immune system surviving after conditioning, i.e. incomplete ablation. This is, however, dosage-dependent. Transplantation of the HSCs should then lead to engraftment and repopulation of the immune system, which is demonstrated by the line graphs. Following high-intensity regimens, the cell counts are at near-0 prior to engraftment and the engrafted cells only repopulate the immune system. Following the other regimens, engrafted cells may compete with the remaining immune cells and then out-compete and predominate the old T cell clones. Initially, however, the TCR repertoire is restricted due to the destruction of T cells, and therefore, T cell diversity is low. Early changes (within 1 year) include the production of de novo immune cells—namely neutrophils, NK, CD8+ T cells and B cells—and/or perhaps repopulation of “old” circulating immune cells, with the later (in around 2–4 years) occurrence of thymic activation or rebound then allowing for the production of a new, more diverse TCR repertoire that is no longer autoreactive or does not allow for the expansion of autoreactive clones

Briefly, the aHSCT procedure involves five stages: pre-transplant optimisation (i.e. identification of co-morbidities, infection prophylaxis, and, e.g. admission for pre-hydration); stem cell mobilisation; conditioning (i.e. myelo- or lymphoablation); stem cell reinfusion; post-transplant supportive care (Fig. 1). The conditioning regimens are variable and considered to be myeloablative or lymphoablative/non-myeloablative (Fig. 1). Near-complete or complete destruction of the bone marrow is considered myeloablative and requires the use of high-dose total body irradiation (TBI) or busulfan. A transplanted graft is necessary for these patients, i.e. autologous recovery will not occur [37]. According to the European Society for Blood and Marrow Transplantation (EBMT), the intermediate-intensity regimens can be described as myeloablative or lymphoablative and may include low-dose TBI or busulfan, although, at least in the context of aHSCT for MS, predominantly include BEAM (a combination of carmustine (**B**CNU), **e**toposide, cytarabine (**A**ra-C) and **m**elphalan) with anti-thymocyte globulin (ATG) (BEAM + ATG) for intermediate myeloablative intensity and cyclophosphamide (CY) and ATG (CY + ATG) or CY and alemtuzumab (ALEM) (CY + ALEM) for intermediate lymphoablative intensity [38]. Again, according to the EBMT, low-intensity regimens include chemotherapy without the addition of antibody therapy, i.e. ATG. Note that BEAM regimens used without ATG have, therefore, been considered as low intensity in this review but BEAM was used without ATG in very few trials. These regimens induce cytopenia and some, to a lesser degree, ablate bone marrow, depending on dosage, but patients do not *require* transplantation.

When first suggested by Burns et al. as a treatment for MS in 1995, aHSCT was proposed to be reserved for patients with progressive disease at onset and was considered an end-stage therapeutic option [39]. Indeed, the first clinical trials included severely affected patients with progressive forms of MS, for whom the protocol of high-dose conditioning followed by aHSCT demonstrated encouraging results [40, 41]. Subsequent clinical trials and observational or retrospective studies that assessed the efficacy of aHSCT using EDSS score, of which there are to the authors’ knowledge approximately 46, have included low-, intermediate- and high-intensity conditioning regimens. Initially, cohorts of patients with progressive forms of MS were included, and soon after mixed cohorts that included those with relapsing forms, with more recently just or mostly RRMS patients being included. Over time, it has become clearer that perhaps the patients who benefit most from aHSCT are those that have RRMS, a lower EDSS score and a shorter disease course—younger age likely also contributes, but all trials assessed young (a median/mean of < 45 years old) patients and there is, therefore, little evidence to support the safety and efficacy of aHSCT in older patients with MS. Despite the accumulative high-quality clinical research over the past 20 years, the heterogenous transplantation regimens, patient populations and description of outcomes do not allow for a robustly evidenced consensus regarding how, when and whom to treat, as well as hinder our understanding of the mechanism of action of aHSCT.

This review, therefore, aims to provide a clear overview of clinical trials treating MS patients with aHSCT, stratified based on conditioning regimen intensity as per the EBMT [38] and the type of study, with the applicable Oxford Centre for Evidence-Based Medicine (OCEBM) Level of Evidence. In addition, the occurrence of late-onset autoimmune disease following aHSCT will be explored. Of note, the reporting of EDSS score has been used most often to compare outcomes and describe study efficacy in this review as, following screening of the articles, it became clear that EDSS was almost always given as an outcome in clinical trials as well as a baseline parameter, thereby serving as a common denominator among otherwise highly heterogeneous articles.

## Overview of aHSCT studies

A total of 2574 patients have been included in either clinical trials or retrospective or observational studies with EDSS as a reported outcome; however, considering, for example, that some patients described in the observational studies likely cross over with those described in the clinical trials, that value may well be an overestimate. 831 (32.3%) patients have been included in 28 clinical trials and 1743 (67.7%) in 18 retrospective or observational studies. As the mean or median age (mAge), duration of MS (mDMS), EDSS at baseline (mbEDSS) and follow-up (mFU) were given variably as either median or mean values among the studies, it is not possible to evaluate the overall median for these data points. These parameters have, however, been listed in Tables 1, 2, 3, 4, 5 and 6, which provide an overview of the studies grouped according to conditioning regimen and study type. The details of each conditioning regimen are given in Supplementary Data 1, Conditioning Regimens. In addition, outcome reporting was heterogeneous with, for example, progression-free survival (PFS) being the most frequently reported measure but given only by 24 (52.2%) of studies. Duration of follow-up was also varied, with a range of 6 months to 11.3 years, making comparison of outcomes challenging.

**Table 1:** Non-randomised, uncontrolled clinical trials. OCEBM level of evidence 2b

Authors	Year published	Country	Phase	No. of patients	Age	MS duration (y)	Type of MS	EDSS (baseline)	Follow-up (y)	EDSS (final)	PFS (y)	Death due to aHSCT
Intermediate-intensity, myeloablative												
Nash et al	2017	USA	II	24	37	4.9	RRMS	4.5	5.2	62% improved, 21% stable	5 y: 91.3%	0
Moore et al	2019	Australia	II	35	37	6.9	RRMS (57%), SPMS (43%)	6	3	44% improved	3 y: 88% (RRMS)	0
Shevchenko et al	2012	Russia	II	95	34.5	–	RRMS (44%), SPMS (37%), PPMS (16%), PRMS (3%)	3.5	3.8	80% improved or stable	5 y: 92% early; 5 y: 73% conventional/salvage	0
Saiz et al	2004	Spain	I	14	32.3	14.9	RRMS (36%), SPMS (64%)	6	3	28% improved, 57% stable	3 y: 85.7%	0
Saccardi et al	2005	Italy	II	19	36	12	SPMS (79%), RRMS (21%)	6.5	3	58% improved, 26% stable	6 y: 95%	0
Capello et al	2005	Italy	II	21	36	12	SPMS (81%), RRMS (19%)	6.5	2	95% improved or stable	–	0
Hamerschalk et al	2010	Brazil	I	20	42	5.3	SPMS (86.7%), PPMS (9.5%), RRMS (4.8%)	6.5	3	44.4% worse	–	3
Kozák et al	2001	Czech Republic	I	10	39	–	SPMS	6.5	0.8	40% improved, 50% stable	–	0
Mancardi et al	2001	Italy	I	10	35.5	12	SPMS	6.5	1.3	100% improved or stable	–	0
Fassas et al	1997	Greece	I/II	15	–	10	PMS	6	0.5	47% improved, 47% stable	–	0
High intensity												
Atkins et al	2016	Canada	II	24	34	5.8	RRMS (50%) SPMS (50%)	–	6.7	70% stable	–	1
Nash et al	2003	USA	I	26	41	7	SPMS (65%), PPMS (31%), RRMS (4%)	7	12	8% improved, 54% stable	–	1
Samijn et al	2006	The Netherlands	I	14	35	5	SPMS	6	3	14% improved, 21% stable	–	0
Burt et al	2003	USA	I/II	21	40	7	SPMS (66%), PRMS (28%), RRMS (4%)	7	2.6 (EDSS > 6), 1 (EDSS < 6)	4% improved, 19% stable	–	0

**Table 2:** Randomised, controlled clinical trials. OCEBM level of evidence 1b

Authors	Year published	Country	Phase	No. of patients	Age	MS duration (y)	Type of MS	EDSS (baseline)	Follow-up (y)	EDSS (final)	PFS (y)	Death due to aHSCT
Intermediate-intensity, myeloablative												
Mancardi et al	2015	Italy and Spain	II	9 (21 total)	36	10.5	SPMS (78%), RRMS (22%)	6.5	4	57% progressed	–	0
Intermediate-intensity, lymphoablative												
Burt et al	2019	USA, UK, Sweden, Brazil	III	55 (110 total)	35.6	4.7	RRMS	3	2	94.5% stable or improved	Too few events	0

**Table 3:** Non-randomised, controlled clinical trial. OCEBM level of evidence 2b

Authors	Year published	Country	Phase	No. of Patients	Age	MS Duration (y)	Type of MS	EDSS (baseline)	Follow-up (y)	EDSS (final)	PFS (y)	Death due to aHSCT
Intermediate-intensity, myeloablative												
Mariottini et al.	2019	Italy	II	11 (52 total)	35	10.5	RRMS	3.25	3	44.4% improved	–	0

**Table 4:** Non-Randomised, uncontrolled clinical trials. OCEBM level of evidence 2b

Authors		Year published	Country	Phase	No. of patients	Age	MS duration (y)	Type of MS	EDSS (baseline)		Follow-up (y)		EDSS (final)		PFS (y)		Death due to aHSCT	
Intermediate-intensity, lymphoablative																		
Burt et al		2009	USA	I/II	21	33	5	RRMS	3.1		3.1		90.5% improved, 9.5% stable		3 y: 100%		0	
Curro et al		2015	Italy	I	7	28	6.5	RRMS	6		5		14% improved, 29% stable		–		0	
Giedraitiene et al		2020	Lithuania	II	24	37.8	8.6	RRMS	5.9		2		23.1% improved, 76.9% stable		–		0	
Hamerschalk et al		2010	Brazil	I	21	41	4.7	SPMS (75%), RRMS (15%), PPMS (10%)	6.5		2		30% worse		–		0	
Cull et al		2017	Australia	I	13	44.8	12.5	SPMS (77%), PPMS (23%)	7.2		3		69% stable		3 y: 69%		0	
Dayama et al		2020	India	I/II	20	31.5	–	SPMS (55%), RRMS (45%)	5.5		0.7		36.8% improved (66.6% of RRMS cohort)		1 y: 100%		0	
de Paula et al		2015	Brazil	I	16	38	8.8	SPMS (50%), RRMS (37.5%), PPMS (12.5%)	5		2		75% improved or stable		–		0	
Low intensity																		
Su et al	2006		China	I	15	36	3	SPMS		6		2.9		40% improved, 27% stable		4.1 y: 63.8%		0
Xu et al	2006		China	I	22	35.5	3	SPMS		6		3.3		59% improved, 18% stable		4.9 y: 77%		0

**Table 5:** Heterogeneous conditioning regimens. OCEBM level of evidence 2b

Authors	Year published	Country	Regimen	No. of patients	Age	MS duration (y)	Type of MS	EDSS (baseline)	Follow-up (y)	EDSS (final)	PFS (y)	Death due to aHSCT
Clinical trials (Phase I and I/II)												
Ni et al	2006	China	High (4.8%), intermediate myeloablative (95.2%)	21	37	3.8	SPMS (89%), PPMS (11%)	7.5	3.5	44% improved, 44% stable	3.5 y: 75%	0
Fassas et al	2002	Multi-centre	High (24%), intermediate myeloablative (47%), intermediate lymphoablative (13%), low (16%)	85	39	7	SPMS (70%), PPMS (26%), RRMS (4%)	6.5	1.3	14% improved	3 y: 63% (PPMS), (78%) SPMS/RRMS	5
Fassas et al	2011	Greece	High (28.6%), intermediate myeloablative (42.9%), low (28.6%)	35	40	7	SPMS (54%), PMS (11%), PPMS (31%), RRMS (2%)	6	11.3	6% improved, 20% stable	15 y: 25%	2
Observational and retrospective studies												
Frau et al	2018	Italy	Intermediate lymphoablative (77.8%), low (22%)	9	38	10	56% RRMS, 22% SPMS, 11% PPMS, 11% PRMS	5.3	11	22% improved, 22% stable	–	0
Muraro et al	2017	Multi-centre (25)	High (18.8%), intermediate myeloablative (38.8%), intermediate lymphoablative (22.8%), low (19.6%)	281	37	6.8	SPMS and PPMS (77.5%), RRMS and PRMS (22.4%)	6.5	6.6	Possible reduction in disability accrual in RRMS group	5 y: 45% (RRMS 75%)	8
Boffa et al	2021	Italy	Intermediate myeloablative (74.8%), intermediate lymphoablative (12.9%), low (12.5%)	210	34.8	11	RRMS (58%), SPMS (41%), PPMS (1%)	6	6.2	Mean EDSS change per year − 0.09 (RRMS). Stable in PMS	–	3
Burman et al	2014	Sweden	Intermediate myeloablative (81.3%), intermediate lymphoablative (18.8%)	48	31	6.3	83% RRMS, 10% SPMS, 4% PPMS, 2% PRMS	6	4	2 y: 3 (5.5) (RRMS), 2 y: 6.5 (6.5) (PRMS)	5 y: 77%	0
Tolf et al	2019	Sweden	Intermediate myeloablative (90%), intermediate lymphoablative (10%)	10	27	2.3	RRMS	6.5	10	100% improved	–	0
Das et al	2021	Multi-centre	Intermediate myeloablative (?), intermediate lymphoablative (?)	20	–	5	“Aggressive” MS	–	2.5	65% stable, 25% improved	–	0
Nicholas et al	2021	UK	Intermediate myeloablative (0.8%), intermediate lymphoablative (99.2%)	120	42.3	8.9	48% RRMS, 52% PPMS or SPMS	6	1.75	65% stable	4 y: 65%	3

**Table 6:** Retrospective and observational studies. OCEBM level of evidence 2b

Authors	Year published	Country	No. of patients	Age	MS duration (y)	Type of MS	EDSS (baseline)	Follow-up (y)	EDSS (final)	PFS (y)	Death due to aHSCT
Intermediate-intensity, myeloablative											
Casanova et al	2017	Spain	31	37	9.5	71% RRMS, 29% SPMS	5	8.4	60% improved, 40% stable (RRMS); 78% progression (SPMS)	12 y: 100% (RRMS)	0
Häußler et al	2021	Germany, France	19	35.1	5.4	63% RRMS, 16% PPMS, 21% SPMS	4.5	4.9	35.7% improved	–	0
Mancardi et al	2012	Italy	74	35.7	11.2	45% RRMS, 55% SPMS	6.5	4	> 7 y: 17% stable, 27% improved	5 y: 66%	2
Krasulova et al	2010	Czech Republic	26	33	7	42% RRMS, 58% SPMS	6	5.5	–	3 y: 84.4% (RRMS), 3 y: 60% (SPMS)	0
Mariottini et al	2021	Italy	26	37	9	SPMS	6	8.3	38% worse	10 y: 30%	0
Mariottini et al	2022	Italy	31 (93 total)	39.3	13.7	SPMS	5.9	8.3	55% worse	5 y: 70%	0
Intermediate-intensity, lymphoablative											
Zhukovsy et al	2021	Sweden	145	30	6.4	RRMS	3	2.8	Improved by 1 on average	–	0
Kvistad et al	2020	Norway	30	30.8	5.2	RRMS	3	2.2	43% improved, 50% stable	–	0
Burt et al	2015	USA	151	36	5.1	RRMS	4	2	52% improved	4 y: 87%	0
Burt et al	2021	USA	507	37	6	82% RRMS, 18% newly diagnosed SPMS	4	3	5 y: 2.19 (3.87) (RRMS), 4 y: 4.72 (5.09) (SPMS)	4 y: 95% (RRMS), 4 y: 66% (SPMS)	1
Low intensity											
Comini-Frota et al	2019	Brazil	5	29	11	RRMS	5.7	9	80% improved, 20% stable	–	0

## Clinical trials: myeloablative

### Non-randomised, uncontrolled clinical trials

The majority of clinical trials are non-randomised and uncontrolled in this field of research, of which 14 used myeloablative conditioning regimens of either high- (4 trials, [42–45]) or intermediate-intensity (10 trials, [40, 46–54]) regimens (Table 1). All trials were phase I, II or I/II.

The high-intensity regimens mostly included SPMS patients with few RRMS, with the 2016 Atkins et al. trial as the exception, including 50% RRMS and 50% SPMS [45]. Patients who were included generally had active disease with progression within the year prior to aHSCT and had trialled at least 1 DMT. The mbEDSS ranged from 6 to 7. Interestingly, Atkins’ trial including the most RRMS patients also demonstrated the best outcomes of all high-intensity regimens in terms of EDSS at last FU. Specifically, stable disease was observed in 70% of patients, whereas the 2003 trial by Nash et al., 2003 trial by Burt et al. and 2006 trial by Samijn et al. described 54%, 19% and 21% of patients being stable at last follow-up, respectively [42–44]. With regard to improvement in EDSS, the highest proportion of patients improved at last follow-up was reported by Samijn et al. at 14%, then Nash et al. at 8% and Burt et al. at 4%. However, each study had a different mFU duration (Table 1). Nash et al. observed patients for the longest time, with a mFU of 12 years. These data demonstrate that for predominately SPMS-containing patient cohorts, with significant proportions of PRMS and PPMS patients in the Nash et al. and Burt et al. trials, respectively, long-term improvement or stability of EDSS is possible. Heterogeneous reporting, patient cohorts and mFU as well as these trials being non-randomised and uncontrolled with a total of 85 patients included does not, although demonstrating promising outcomes in some cases, provide a strong evidence base for offering patients with progressive forms of MS high-intensity aHSCT, particularly given that this type of transplant is associated with the highest occurence of death (see “Adverse events, mortality and autoimmune disease”). Data for patients with RRMS for this conditioning regimen are slighter still with very little evidence available to support offering these patients high-intensity conditioning regimens.

Non-randomised, uncontrolled trials including intermediate-intensity myeloablative conditioning regimens also mostly included patients with progressive forms of MS, namely SPMS (Table 1). Mancardi et al. included a cohort of 10 patients with SPMS, 100% of whom were improved or stable with regard to EDSS at a mFU of 1.3 years [54]. These patients had a mDMS of 12 years, mAge of 35.5 years and mbEDSS of 6.5, which would imply that this cohort is very much burdened by disease and so the impressive findings are encouraging, although the mFU is short. As demonstrated in Table 1, the mFU was quite short for all clinical trials, save for the 2017 Nash et al. trial, which included RRMS patients only and provided strikingly promising data, with the longest mFU of 5.2 years [46]. For these patients, a 5-year PFS of 91.3%, 62% improvement in EDSS and a stable EDSS score in 21% was observed at last follow-up. Long-term PFS for a smaller proportion of the initial patient cohort was given by Shevchenko et al. and Saccardi et al. (Table 1) [48, 50]. Regarding the former, PFS was 92% at 5 years for patients who had received early transplantation and 73% for those who received aHSCT on conventional or salvage grounds. This is one of few trials to demonstrate that the hit hard and early strategy does provide better outcomes, with a cohort including 44% RRMS, 37% SPMS, 16% PPMS and 3% PRMS patients, and with a mFU of 3.8 years. Saccardi et al. noted a 6-year PFS of 95% for a cohort of predominantly SPMS patients, as well as encouraging EDSS outcomes at last follow-up with 84% improved or stable. These 10 trials provide information on a total of 224 patients, with encouraging PFS rates and EDSS scores at last follow-up following intermediate-intensity myeloablative conditioning for a diverse population of MS patients.

### Randomised, controlled clinical trials

The only randomised, controlled trial for myeloablative aHSCT, performed by Mancardi et al., was of intermediate intensity, had mitoxantrone as the comparison arm, and included a total of 21 patients, of whom 9 underwent a transplantation [55] (Table 2). The majority of patients had SPMS and, after a mFU of 4 years, 57% of patients experienced worsened disability. Patients who received aHSCT did have significantly fewer (79%) MRI lesions compared to mitoxantrone and a reduced annualised relapse rate, but with no difference in disability progression between groups. Few conclusions may be drawn from such a small cohort, despite an OCEBM Level of Evidence of 1b.

### Non-randomised, controlled clinical trials

Mariottini et al. designed a non-randomised, controlled trial to establish the efficacy of aHSCT following cessation of natalizumab in 52 RRMS patients, of whom 11 underwent transplantation with an intermediate-intensity regimen [56] (Table 3). The remaining patients received a DMT. After a mFU of 3 years, 44.4% of patients had an improved EDSS score and NEDA was reported in 54.4% compared to 11.5% of the control group. Again, although encouraging, it is challenging to confidently base treatment decisions on such a small cohort of patients.

## Clinical trials: lymphoablative

### Non-randomised, uncontrolled clinical trials

Nine of the non-randomised, uncontrolled trials included low-intensity (two trials, [57, 58]) or intermediate-intensity lymphoablative regimens (seven trials, [52, 59–64]) (Table 4). All trials were phase I, II or I/II.

Again, the majority of trials included patients with SPMS, although three included RRMS patients only. Of these three, which all used intermediate-intensity regimens, the 2009 trial by Burt et al. observed a 90.5% improvement in EDSS at last follow-up, mFU was 3.1 years, with 9.5% of patients remaining stable [59]. PFS was 100% and NEDA 62% at 3 years. The 21 patients in this cohort had a mAge of 33, mbEDSS of 3.1, mDMS of 5 years and had experienced treatment failure on DMT. Further significant improvements were seen in neurological rating scale score, the paced auditory serial addition test, the 25-foot walk test and Short Form-36 score (SF-36). In contrast, the 2 other studies including RRMS patients only, for whom DMT had also not been effective, were by Curro et al. and Giedraitiene et al. and observed EDSS improvements of 14% (29% were stable) and 23% (77% were stable), respectively, but with an almost twice as high mbEDSS compared to the Burt et al. cohort [60, 61]. Interestingly, Giedraitiene et al. found that patients with a lower baseline EDSS had better outcomes. These data add evidential weight to the EBMT guidelines that patients with a lower EDSS score are the optimal candidates for aHSCT but included only small patient cohorts. The other three intermediate-intensity regimen trials included mainly SPMS patients, with Cull et al. providing the longest mFU of 3 years and describing disease stability in 69% of patients [62]. Although Dayama et al. also reported encouraging results, the mFU was 0.7 years, and therefore too short to provide meaningful data [63]. The intermediate-intensity regimens were assessed in trials that enrolled a total of 106 patients, with some encouraging data and an interesting suggestion that lower EDSS score correlates with improved outcomes.

The two low-intensity regimens included in this section used chemotherapy-only conditioning regimens, i.e. did not use ATG, and were, therefore, classified as low intensity as per the EBMT guidelines [38, 57, 58]. However, both trials used the BEAM regimen, which is often considered an intermediate-intensity regimen. Both studies report encouraging results for SPMS only patient cohorts, with Su et al. observing a 4.1-year PFS of 63.8% and Xu et al. a 4.9-year PFS of 77% (Table 4). Most trials use ATG and it is noteworthy that these two trials reporting encouraging outcomes do not.

### Randomised, controlled clinical trials

The 2019 Burt et al. randomised, controlled trial using an intermediate-intensity lymphoablative regimen included RRMS patients only, a total of 110, of whom 55 received a transplantation (Table 2) [65]. Of note, 31 patients in the DMT group crossed over to receive aHSCT due to worsening disability at 1 year. It should, however, be noted that the DMT group included a high proportion of patients treated with glatiramer acetate or the IFN-β drugs and few with higher intensity medications such as natalizumab or fingolimod. Progression occurred in 3 of the aHSCT patients and 34 of the DMT control group, with the EDSS in the aHSCT group at a mFU of 2 years stable or improved in 94.5%. NEDA at 5 years was 78.5% in the aHSCT group compared to 2.97% in the DMT group. These data quite clearly indicate better long-term outcomes for patients with RRMS receiving intermediate-intensity lymphoablative aHSCT compared to DMT, albeit the less effective DMTs, in a relatively large patient cohort.

## Clinical trials: heterogeneous conditioning regimens

The three phase I or I/II trials including heterogeneous conditioning regimens and heterogeneous patient populations are difficult to critically analyse [66–68] (Table 5). Of particular interest in this cohort is the trial by Fassas et al., who was the first to perform aHSCT in patients with MS in 1997 and provides the longest mFU data of all studies (11.3 years) [67]. The 15-year PFS in this cohort was 25%, with patients that had a lower baseline EDSS demonstrating better outcomes.

## Retrospective and observational studies: myeloablative

All retrospective and observational studies used intermediate-intensity myeloablative regimens [69–74] (Table 6). A remarkable 100% 12-year PFS in the RRMS cohort has been reported by Casanova et al., with the RRMS cohort demonstrating better outcomes than those with SPMS [69]. For these patients, mbEDSS was 5, mAge 37, at least 1 DMT had been trialled and the mDMS was 9.5 years. At last follow-up, mFU was 8.4 years, 60% of the patients with RRMS had improved and 40% were stable, which is in stark contrast to 77.8% of the SPMS cohort experiencing disability progression. In addition, Casanova et al. observed that poor response to aHSCT was predicted by high EDSS score at baseline. Again, demonstrating better outcomes for RRMS compared to SPMS patients is Krasulova et al., with a 3-year PFS of 84.4% and 60%, respectively. Krasulova et al. also observed better PFS outcomes in patients with disease duration of less than 5 years and patients under 35 [73]. Of note, Mancardi et al. found that a greater improvement in EDSS was associated with RRMS patients compared to those with SPMS, age below 40 and disease duration of less than 5 years [72]. Mariottini et al. report a 10-year PFS and NEDA of 30% for their SPMS cohort, further demonstrating that very long-term outcomes are better for RRMS patients [71]. Of note, Häußler et al. reported a 10-year NEDA of 62% following aHSCT and demonstrated that patients receiving aHSCT had better outcomes than those receiving ALEM [70]. Mariottini et al. compared the BEAM + ATG regimen for aHSCT with CY 0.75 g/m2 BSA given monthly for the first year of treatment, then every second month in the second year and quarterly in the third year [74]. aHSCT was found to be more effective at preventing relapse than CY but the effect on long-term disability progression was similar between groups.

## Retrospective and observational studies: lymphoablative

Six studies included lymphoablative regimens, with one being of low [75] and four of intermediate intensity [76–79]—all these studies included mainly RRMS patients (Table 6). Of these 4, and indeed of the entire 46 studies described in this review, the largest patient cohort was provided by Burt et al. and reported recently in this journal, which included 507 patients with RRMS (82%) and newly diagnosed SPMS (18%) [79]. In keeping with the findings from the myeloablative retrospective and observational studies, Burt et al. described a PFS at 4 years of 95% for patients with RRMS and 66% for those with SPMS. At 5 years, mEDSS had improved to 2.19 from 3.87 in the RRMS patients and at 4 years to 4.72 from 5.09 in the SPMS patients. Of note, Zhukovsy et al. demonstrated in their cohort of 145 RRMS patients that EDSS score improved by 1 on average with a mFU of 2.8 years, corroborating with the findings of Burt et al.’s 2021 data [76]. Again, similar to the findings of the myeloablative regimens, namely Häußler et al. [70], Zhukovsy et al. reported superiority of aHSCT compared to ALEM. The data for RRMS patients receiving intermediate-intensity lymphoablative aHSCT reflects outcomes from a remarkable 833 patients and is similar across studies, providing supportive evidence for this regimen in patients with RRMS. The low-intensity regimen also demonstrated very encouraging data but included only 5 patients [75].

## Retrospective and observational studies: heterogeneous conditioning regimens

Retrospective and observational studies containing data with heterogeneous regimens are included in Table 5 [80–86]. Muraro et al. analysed data from 281 patients and again demonstrated better PFS in RRMS patients and in patients with a lower EDSS score at baseline [82]. In addition, Muraro et al. found that younger age and fewer prior immunotherapies were associated with better outcomes. Boffa et al. included 210 patients and observed a mean EDSS change per year of -0.09 in the RRMS cohort compared to EDSS being stable in the progressive MS cohort [80], which was similar to Burman et al., who found that at 2 years the RRMS cohort mEDSS had improved to 3 from 5.5 and in the PRMS cohort had remained stable 6.5 [84]. Also of note in this group of studies is that Tolf et al. reported 100% of the 10 patients with RRMS had improved EDSS scores after a mFU of 10 years [85]. Although heterogenous, 698 patients were included and results from this large cohort reflect the data from the other retrospective and observational studies.

## Key points from the clinical trial data and EBMT guidance

High-quality evidence in the form of randomised, controlled trials is needed to compare the efficacy of aHSCT to the currently available highly effective DMTs and thereby draw confident conclusions regarding the risk vs. benefit of transplantation in MS. However, the clinical trials discussed in this review provide critical guidance regarding patient selection and transplantation protocol, with further advice for experienced centres considering aHSCT also provided by the EBMT [38]. Data from the trials included in this review demonstrate that younger age, shorter duration of disease, relapsing–remitting disease course and lower baseline EDSS have better outcomes. The EBMT advise that aHSCT may be considered for patients aged 45 or younger with highly active RRMS, an EDSS of 5.5 or less and a duration of disease of 10 years or less, who have failed at least one first-line DMT [38]. In addition, they suggest that patients with aggressive MS, i.e. with a rapidly accelerated disease course, may be considered for aHSCT prior to completion of the full course of a first-line DMT [38]. Following patient selection, an extensive pre-aHSCT evaluation of fitness for transplantation including echocardiography, electrocardiogram, pulmonary function testing, blood testing including an infection screen, and psychological evaluation must occur. In addition to this, counselling of patients regarding the risks of transplantation is required, particularly death, serious adverse events, infection, autoimmune disease, and infertility. Patients who meet the inclusion criteria may, therefore, be excluded following evaluation of fitness for transplantation. With regard to transplantation protocol, the data from this review suggest that high-intensity conditioning is associated with an increased occurrence of death without obvious benefit to efficacy. Low-intensity regimens were rarely used. Conditioning using CY + ATG and BEAM + ATG appear to be the most widely used, with both demonstrating promising efficacy and both suggested as the most evidenced options by the EBMT [38]. CY + ATG appears to offer a better safety profile from the data in this review, particularly with Burt et al.’s real-world cohort of 507 patients providing encouraging outcomes and safety data. It should also be noted that the promise of aHSCT for patients with SPMS remains to be fully clarified, with the EBMT suggesting that patients with active inflammation and clear disability progression may be the best candidates, but this should be considered in the context of a clinical trial [38]. In support of this is the very recent retrospective study by Mariottini et al. that compared SPMS patients treated with aHSCT (using BEAM + ATG) to those treated with CY alone, i.e. immunosuppression only, and demonstrated that aHSCT was far superior to CY at suppressing inflammation, e.g. reducing relapses, but had a minimal effect on disability progression [74].

## Adverse events, mortality and autoimmune disease

The expected side effects of stem cell transplantation were noted in most studies, in which adverse events were described, e.g. viral reactivation, bacterial infection, and febrile neutropenia. Of particular interest is the reporting of death and the occurrence of autoimmune disease, as these outcomes are perhaps less expected than the more common side effects and relevant to the discussions had with patients prior to aHSCT. In addition, this section will discuss the potential for neurotoxicity and secondary malignancy using these agents, as well as implications on fertility, vaccination, and immunity. It should be noted that both the very earliest and the most recent trials have been included in this review. The treatment-associated mortality of aHSCT has improved in recent years [35], which should be kept in mind when considering these outcomes. Of interest, Mancardi et al. describe that, in Europe, the mortality due to aHSCT was 7.3% between 1995 and 2000 and 1.3% between 2001 and 2007 [87].

### Death

The percentage of total patients who underwent transplantation and died as a consequence, with deaths related to conditioning regimen, was 2.4% following a high-intensity regimen (2 deaths, 85 patients), 1% following myeloablative intermediate-intensity conditioning (5 deaths, 491 patients), 0.1% following lymphoablative intermediate-intensity conditioning (1 death, 1065 patients) and 0% following low-intensity conditioning (0 deaths, 42 patients). The two deaths following high-intensity transplant were due to massive hepatic necrosis (conditioning regimen: 14.9 mg/kg busulfan) [45] and Epstein–Barr virus (EBV)-related post-transplantation lymphoproliferative disorder (associated with a change from horse-derived to rabbit-derived ATG) [42]. Following myeloablative intermediate-intensity conditioning, five deaths were due to cardiac toxicity in one patient (1), sepsis (1) and alveolar haemorrhage (1) [52], engraftment failure, subsequent *Actinomyces* spp. infection and disseminated intravascular coagulation (1) [72] and encephalopathy of unknown aetiology (1), with all deaths following BEAM + ATG [72]. The single death in the intermediate-intensity lymphoablative cohort was due to hospital-acquired legionella pneumonia [79]. It is worth noting that in their CY control group, Mariottini et al. described one death due to pneumonia 17 years following treatment and one death due to intracerebral haemorrhage with thrombocytopenia following splenectomy for a splenic infarct 8 years following treatment [74].

In the three clinical trial cohorts of patients receiving heterogenous conditioning regimens, seven transplant-related deaths were reported by Fassas et al., of which five occurred during the clinical trial for which results were published in 2002 and two in the long-term follow-up report of the 1995 clinical trial, published in 2011 [66–68]. The five deaths reported in 2002 were due to cardiac toxicity (1, BEAM only regimen), cerebral aspergillosis (1, BEAM + ATG), septicaemia (1, BEAM + ATG), influenza pneumonitis (1, busulfan (Bu) + CY + ATG) and postoperative pneumococcal sepsis (1, Bu + CY + ATG), the latter of which occurring 19 months after transplant. Including these deaths in the mortality data increases the percentage of total patients dying as a consequence of high-intensity conditioning to 3.8% (4 deaths, 105 patients), myeloablative, intermediate-intensity conditioning to 1.4% (7 deaths, 501 patients), and low-intensity conditioning to 1.8% (1 death, 56 patients). The two deaths reported in 2011 were due to pulmonary haemorrhage associated with post-transplant-onset acquired haemophilia A (1) and aspergillosis (1), occurring in the BEAM cohort and the Bu cohort, although it is unclear which death was associated with which conditioning regimen. Of note, Ni et al. reported no deaths associated with transplantation but acknowledged one patient dying of severe pneumonia at 4.5 months following transplant and a further patient suffering from varicella-zoster hepatitis at 15 months post-transplant in a cohort of 21 patients receiving either a high- or intermediate-intensity conditioning regimen [68].

Muraro et al. analysed factors associated with worse overall survival in a multi-centre, heterogenous cohort of patients included in an observational study, within which 8 deaths were reported, and found that higher baseline EDSS was significantly associated with a higher risk of death over time [82]. Combined with the evidence that suggests patients with a lower EDSS score have better outcomes in terms of disease progression, this further supports the patient selection parameters suggested by the EBMT [38, 82]. In addition, Muraro et al. observed that, among the patients who died, progressive MS and high-intensity conditioning regimens were overrepresented compared to the frequency of these factors in the entire cohort [82]. In this study, we also found that high-intensity conditioning regimens were associated with the highest percentage of patient deaths, which is unsurprising given that this treatment includes aggressive myeloablative agents. Boffa et al. reported the deaths already reported by Mancardi et al. but considered the death due to pulmonary thromboembolism followed by syncope and head trauma 56 days after AHSCT as transplantation-related, which Mancardi et al. had reported, following review by an independent committee, as not related to transplantation [72]. Nicholas et al. also reported three deaths in a cohort who received heterogenous regimens, which were due to cardiac arrest secondary to recent pulmonary oedema the day before transplantation (1), cardiac arrest secondary to electrolyte abnormalities (1), and acute respiratory distress syndrome (ARDS) secondary to a chest infection and sepsis (1) [86]. Regarding potentially life-threatening side effects unrelated to infection, Giedraitiene et al. reported a case of ARDS during the administration of conditioning with CY + ATG that resolved with glucocorticoid therapy [61] and Mancardi et al. reported life-threatening dyspnoea, bradycardia, and hypoxemia secondary to ATG [55]. Of note, Ni et al. reported one allergic reaction to CY and a further allergic reaction to ATG in another patient.

### Neurotoxicity

Interestingly, neurotoxicity was rarely reported in the trials considered by this review but is often reported in the context of stem cell transplantation more generally, and agents used in the BEAM regimen, for example, certainly have the potential to be neurotoxic [88–91]. Perhaps this is due to the aHSCT regimens often used in MS, and those recommended by the EBMT [38], using less potent dosages of ablative agents for a relatively short duration as a part of “intermediate-intensity” regimens, as complete myeloablation is not the priority. Transient neurological deterioration was, however, described in five patients was reported by Ni et al., who used both a high-intensity and intermediate-intensity regimen in SPMS and PPMS patients. The encephalopathy-associated death reported by Mancardi et al. is perhaps also of relevance in this context, although no definite cause was reported [72].

### Secondary malignancy

Regarding the risk of secondary malignancy in MS patients undergoing aHSCT, conclusive evidence has not been provided by these trials and further long-term follow-up data are required. Particularly with CY, which has been associated with bladder cancer and haematological malignancies [92]. In the real-world Burt et al. cohort of 507 patients who received CY, the authors specifically mention no incidence of bladder cancer, myelodysplastic syndrome (MDS) or leukaemia and report one case of death secondary to T cell lymphoma 10 years following aHSCT, as well as one death due to colon cancer 3 years following aHSCT [79]. Other reports of secondary malignancy were reported by five studies. Casanova et al. reported two cases of breast cancer and one case of cervical intraepithelial neoplasia grade 2 following BEAM + ATG [69], Mariottini et al. reported a case of MDS 12 years after transplantation with CY + azathioprine [71], Samijn et al. also described a patient who developed MDS and a further patient who developed (EBV)-related post-transplantation lymphoproliferative disorder following TBI + CY + ATG, who was effectively treated with rituximab [44], and Fassas et al. reported a case of prostate cancer 12 years after transplantation in the cohort who received heterogenous conditioning regimens [67]. Mariottini et al. reported one case of myeloproliferative disorder 12 years following aHSCT with BEAM + ATG [74]. Interestingly, the authors also reported Hodgkin lymphoma in once patient at month 10 and renal cancer in one patient at year 15 following treatment in the CY comparison group.

### Infertility

Despite this being a highly relevant side effect of the agents used in these conditioning regimens, fertility outcomes and fertility-conserving measures were rarely mentioned by the studies. Kvistad et al. offered all men and women below 35 in their cohort treated with CY + ATG fertility-conserving treatment and describe 43% of women having persistent symptoms of ovarian failure following transplantation, of which 60% had this confirmed on further diagnostic testing [77]. The oldest woman was 44 and the youngest 25. Häußler et al. mention one case of infertility following transplantation using BEAM + ATG [70]. Further evaluation of fertility outcomes is required to understand the risk of, and risk factors associated with, infertility for this patient cohort; however, fertility and planning for future pregnancy should certainly be a part of counselling for younger patients considering aHSCT.

### Autoimmune disease

Reported autoimmune diseases were hyper- or hypothyroidism, immune thrombocytopenic purpura (ITP), alopecia areata, acquired haemophilia A and arthritis [56, 66, 67, 70, 74, 76, 77, 82–84]. Crohn’s disease was also reported [82, 84]. Das et al., following an intermediate-intensity either myelo- or lymphoablative regimen in patients with aggressive MS, reported the occurrence of autoimmune hypo- or hyperthyroidism in as many as 20% of their cohort [83]. Interestingly, regarding CY + ALEM regimens, Burt et al. reported two patients developing ITP post-ALEM in the 2009 trial [59], and in the 2015 and 2021 cohorts, Burt et al. observed an incidence of post-transplant autoimmune disease of 22.7% and 11.5%, respectively, in the CY + ALEM cohort vs. 6.9% and 2–3%, respectively, in the CY + ATG cohort [79]. Häußler et al. also reported a higher number of patients affected by autoimmune disease in their ALEM cohort [70]. From these data, it would appear, as has been suggested by Burt et al. and Ruck et al., that the risk of autoimmune disease development is increased by using ALEM in the conditioning regimen [79] [Ruck T et al. Alemtuzumab-induced immune phenotype and repertoire changes: implications for secondary autoimmunity. *Brain* (2022). Ahead of publication (manuscript accepted)].

### Vaccination and immunity

Regarding guidance for vaccination following aHSCT, a comprehensive report covering numerous aspects of immune reconstitution following aHSCT for MS was recently published on behalf of the EBMT [93]. The authors describe the loss of immunity following transplantation, as well as persistently poor immune defence against pathogens that persists for several years post-aHSCT. The EBMT guidance is, therefore, to implement a routine vaccination program 3–6 months following aHSCT, with the knowledge that, in the early post-transplantation period, vaccination response may be suboptimal [93].

## Immune reconstitution following aHSCT

It is thought that the expansion of autoreactive T cell populations following the failure of immune tolerance mechanisms is a driving factor in MS pathogenesis [94, 95]. The restoration of self-tolerance via immune reconstitution following aHSCT is, therefore, said to be why such markedly good outcomes are observed in large cohorts of patients that, in a number of cases, persist into the long-term [96] (Fig. 1). Recently, Visweswaran et al. observed the recalibration of relevant pro-inflammatory and immunoregulatory lymphocyte subsets persisting at 36 months following transplantation [36]. The results from studies assessing immune reconstitution following aHSCT should be considered with the knowledge that each investigated a demographically different cohort of patients who had received different transplant protocols. The findings were, therefore, diverse, considering also that different immune system compartments were assessed. Those that were common among articles will be discussed in this section.

Absolute lymphopenia occurs immediately following transplantation and is reported to return to baseline levels from 6 months to 1 year post-aHSCT [47, 62, 82, 96, 97]. There is evidence of an early shift towards anti-inflammatory signalling through decreased IL-21 and 22 with increased CCL2 and CCL4 [98] as well as rapid reconstitution of NK cells post-aHSCT contributing to Th17 suppression [99], with Th17 numbers also observed to decrease by Cull et al. [62]. Visweswaran et al. observed a sustained decrease in the frequency of the Th17 subset at both 24 and 36 months, but with no change in absolute Th17 cell numbers [36]. Of note, Th17 is thought to directly participate in MS-associated neuroinflammation and damage oligodendrocytes [100]. Numerous studies report that CD4+ T cell populations do not return to baseline 2 years post-aHSCT, with altered CD4+ /CD8+ T cell ratios, therefore, persisting in the long term [47, 62, 96, 97, 101]. However, Visweswaran et al. recently described the reduced CD4+ /CD8+ ratio observed in their cohort beginning to normalise by 36 months post-transplantation [36]. Arruda et al. specifically demonstrated that CD4+ central memory (CD4+ _CM_) T cell populations are ablated and both CD4+ and CD8+ effector memory (CD4+ _EM_, CD8+ _EM_) are preserved [96]. The authors concluded that, during the first 2 years, the peripheral T cells remaining after conditioning are the likely predominant contributors to the reconstitution of the T cell pool post-aHSCT. Muraro et al. found that CD4+ _EM_ T cells rose significantly at 6 months then declined towards baseline, whereas CD4+ _CM_ T cell populations steadily decreased during post-transplant follow-up and were significantly decreased at 2 years [102]. A significant proportion of the T cell pool occupied by the CD4+ _CM_ T cell population was then repopulated by naïve CD4+ T cells (CD4+_naïve_), which has also been observed by Hakim et al. [103], that the authors suggested to evidence rejuvenation of the T cell repertoire [102]. A trend for decreased CD8+ _CM_ T cells was also observed by Muraro et al. at 2-year follow-up. However, Abrahamsson et al. did not observe a change in CD4+_naïve_ T cells in the CD4+ subset in their cohort of patients following lymphoablative conditioning—vs. myeloablative in the Muraro et al. cohort—and suggested that immune reconstitution is, in this context, secondary to the expansion of differentiated T cells acquiring effector cell phenotypes [97]. These data highlight that, although each conditioning regimen does demonstrate promising clinical data, the mechanisms of action may well be different and unification of future studies with regard to methodology would allow for better understanding of the mechanism of action of aHSCT in larger patient cohorts.

One of the key aspects contributing to long-term immune reconstitution has been elicited by the study of T cell receptor (TCR) diversity. Muraro et al. provided the initial data in this area of MS research and demonstrated increased clonal diversity of T cells due to de novo TCR rearrangement following aHSCT [102, 104]. The authors suggested that initial immune cell repopulation is dependent on expansion of the autologous graft, with a later (starting from > 1 year) marked increase in CD4+ T cell receptor excision circle (TREC) levels, with TREC levels being a surrogate marker for thymic activity, perhaps indicating that the later clonally expanded, more diverse populations are derived from de novo selected T cells and thymic rebound (Fig. 1). CD8+ TREC levels recovered to baseline but were not increased. Of note, thymic rebound has become an increasingly interesting concept in aHSCT research, and it may be that strategies to enhance thymic rebound would be of benefit to patients following aHSCT as a means of bolstering T cell reconstitution [105]. Harris et al. observed almost complete removal of the pre-existing TCR repertoire following transplant, which was maintained throughout the 2 years of follow-up [101]. Interestingly, the T cell clones present in cerebral spinal fluid (CSF) prior to transplant that were not detectable in the blood did not persist after transplant, and instead > 90% of the intrathecal T cell repertoire was replaced by new clones derived from the peripheral circulation [101]. Harris et al. proposed that TCR repertoire replacement in the CSF could act as a surrogate marker for aHSCT efficacy. In addition, Amoriello et al. suggested that the evaluation of clonal persistence in memory T cell subpopulations may allow for prediction of patient outcomes following aHSCT but that this should be considered in a patient-specific, individualised manner [106].

## Monitoring the efficacy of transplant

It was decided to use EDSS as a marker of efficacy for this review due to this parameter being the most frequently reported across studies. The EDSS also provides centres with perhaps more limited resources an inexpensive and well-evidenced monitoring tool. However, the assessment of disease activity on magnetic resonance imaging (MRI) is a highly sensitive, fundamental aspect of follow-up and should be a part of outcome reporting following aHSCT, particularly regarding evaluation of NEDA. Interestingly, MRI monitoring is not only helpful in assessing progression, but has also demonstrated, in a small number of patients, increased brain atrophy rates in the first 1 to 2 years post-transplantation—atrophy was reported to occur up to 10 times faster compared to pre-transplantation in an early report by Chen et al. [107]. The authors suggested that perhaps resolution of oedema and chemotoxicity may contribute to this finding. Inglese et al. found that progressive atrophy can occur following aHSCT independent of active inflammation on MRI, which may be related to the severe disease phenotype of transplanted patients, the aHSCT procedure, resolution of inflammation and oedema, or persistent demyelination and loss of trophic factors contributing to persistent neuronal death [108]. Few trials included in this review evaluated brain atrophy rate in relation to aHSCT. In a cohort of SPMS patients, Mariottini et al. found a slightly increased atrophy rate post-transplant that later normalised in 55% of patients [71]. Using patient data from the Samijn et al. trial [44] and again in an SPMS cohort, Rocca et al. demonstrated a median decrease in brain volume of 1.92% over the first year following aHSCT, which reduced to 1.35% in the second year then 0.69% in the third year [109]. The authors found that the number of enhancing lesions was significantly correlated with the percentage of brain volume change between baseline and month 12, but not over the second and third years. Samijn et al. reported that the rate of brain atrophy in the third year following transplantation was similar to that of patients with less aggressive forms of MS [44].

To further investigate the occurrence of brain atrophy following aHSCT, it would be of interest to assess for changes to smouldering inflammation and slowly expanding lesions, as well as serum neurofilament light chain (sNfL) levels. Of note Mariottini et al. recently analysed sNfL levels in 38 patients with RRMS or SPMS previously recruited to undergo aHSCT using a BEAM + ATG regimen and compared the data to 22 SPMS, not transplanted, patients and 19 healthy controls [110]. The authors explained that sNfL significantly decreased 24 months following transplantation, particularly in the RRMS cohort, which suggests that aHSCT can induce a durable reduction in inflammation-related axonal damage. As the reduced sNfL level at 24 months was similar to that of the SPMS control cohort, the authors suggested that this reflects resolution of recent inflammatory activity. Interestingly, Mariottini et al. also described a transient increase in sNfL 6 months following transplantation and suggested that this may be due to the toxicity of the chemotherapies or rapid suppression of inflammation causing neuronal damage, or both [110]. However, the authors state that blood samples were not collected shortly after transplant and an early increase in sNfL due to the neurotoxicity of chemotherapy could not be evaluated. Clearly, these biomarkers are of value in this context. Indeed, generally in future studies evaluating aHSCT, these more sensitive monitoring tools would be of great value to outcome data, as well as establishing whether alterations in oligoclonal band (OCB) or glial fibrillary acidic protein (GFAP) production occurs following transplantation, particularly when compared to a control group [111].

## Future clinical trials

Currently, there are seven active studies listed on clinicaltrials.gov, of which five are recruiting. There are two clinical trials comparing aHSCT with alemtuzumab and one further comparing aHSCT with the best-available therapy (BEAT-MS). Three additional studies are assessing aHSCT for the treatment of MS. One trial is assessing the effect of faecal microbiota transplantation after aHSCT in MS. Interestingly, of these studies, AutoMS-Swe will compare the safety and efficacy between the two intermediate-intensity conditioning regimens BEAM-ATG and CY-ATG. If patients are to be treated with aHSCT at an experienced centre outside of a clinical trial, data should be sent to a patient registry, for example the EBMT Patient Registry (https://www.ebmt.org/ebmt-patient-registry).

## Conclusions

There are good data available showing efficacy and safety of intermediate-intensity conditioning regimens in aHSCT for the treatment of MS. Guidelines regarding the suggested candidates and conditioning regimens for transplantation have been provided by the EBMT [38], and this review supports these recommendations. In addition, although there are minimal data available for high-intensity regimens, it does not appear that there is any benefit to choosing these more aggressive treatment options but rather that these approaches are associated with significantly more toxicity. Increased intensity of the conditioning regimen clearly does not necessarily lead to increased efficacy of aHSCT, which is supported by evidence that immune reconstitution occurs without complete myeloablation [62, 96, 97, 112]. Randomised, controlled trials are urgently needed to generate data that clearly indicates aHSCT is superior to other, less aggressive, treatment options available, with three trials comparing aHSCT to either alemtuzumab or the best-available treatment option currently in progress. It will be of interest to note if these trials also observe an increased prevalence of autoimmune disease in their alemtuzumab populations and indeed in the cohort of patients receiving aHSCT, as this appears to be an important outcome parameter that affects a significant proportion of patients in the long term. Why this occurs is certainly a research area of interest, as is exactly how immune reconstitution leads to restored immune tolerance in the patients who benefit from transplantation.

## Supplementary Information

Below is the link to the electronic supplementary material.

Supplementary file1 (XLSX 11 kb)
