# Correction to: The current standing of autologous haematopoietic stem cell transplantation for the treatment of multiple sclerosis

**DOI:** 10.1007/s00415-022-11158-z

**Published:** 2022-05-24

**Authors:** A. G. Willison, T. Ruck, G. Lenz, H. P. Hartung, S. G. Meuth

**Affiliations:** 1 Department of Neurology, Medical Faculty, University Hospital Düsseldorf, Heinrich Heine University, Düsseldorf, Germany; 2 Department of Medicine, University Hospital Münster, Albert-Schweitzer-Campus 1, Münster, Germany; 3 Department of Neurology, Medical University of Vienna, Vienna, Austria; 4 Brain and Mind Center, University of Sydney, Sydney, Australia; 5 Department of Neurology, Palacky University, Olomouc, Czech Republic

## Correction to: Journal of Neurology 10.1007/s00415-022-11063-5

The original version of this article unfortunately contained a mistake in the figures C and D of the following section “3. Conditioning” of the Fig. 1.

The corrected Fig. 1 is given in the following page.

**Fig. 1:**
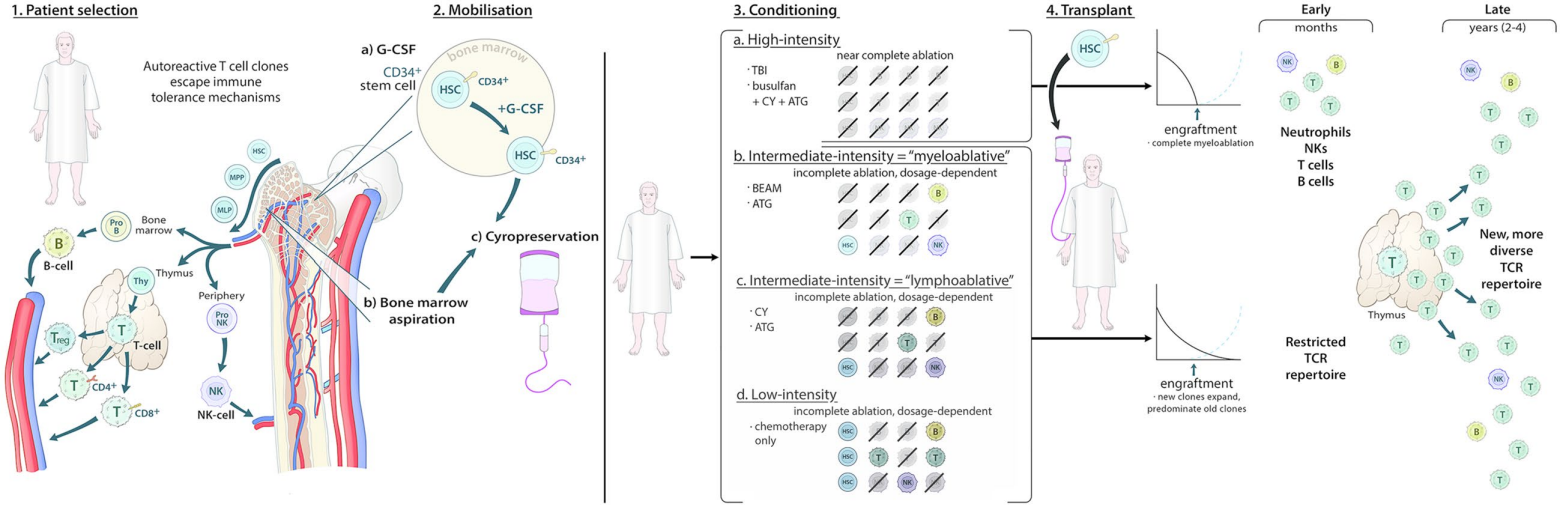
Autologous transplantation and immune reconstitution. The first stage is patient selection, where fitness and suitability for transplant are considered. In patients with MS, autoreactive T cell clones that have escaped immune tolerance mechanisms contribute to pathogenesis by effecting neuroinflammation, along with B cells and natural killer (NK) cells. These cells have common progenitors in the bone marrow, which are—at the earliest—haematopoietic stem cells (HSC), with later stages including multipotent progenitors (MPP) and multilymphoid progenitors (MLP). B cell maturation occurs primarily within the bone marrow. T cell maturation occurs within the thymus, with the bone marrow producing thymocytes that then undergo a complex maturation process within the thymus, producing regulatory T cells (Treg), CD4+ T cells and CD8+ T cells. Treg may also mature peripherally. NK cells begin their maturation process within the bone marrow, which is completed in the periphery. The transplant process then is initiated during mobilisation, where, most commonly in MS, HSC are either extracted peripherally (2a) following G-CSF (often with cyclophosphamide or rarely using cyclophosphamide alone) administration or, less commonly, from the bone marrow proper using bone marrow aspiration (2b). Cells are then cryopreserved (2c). The patient may then undergo conditioning, which may be of four intensities according to the European Society for Blood and Marrow Transplantation: high intensity, using total body irradiation (TBI) alone or in combination with other agents or busulfan with cyclophosphamide and anti-thymocyte globulin (ATG); intermediate-intensity “myeloablative” using BEAM + ATG; intermediate-intensity “lymphoablative” using cyclophosphamide + ATG; low-intensity using chemotherapy-only regimens, i.e. without ATG. For the high-intensity regimens, the conditioning should destroy all remaining immune cells and, therefore, at transplantation the patient has no immune cells being produced or in circulation, which is complete ablation. The other regimens will have varying degrees of immune cell destruction, with cells from the “old” immune system surviving after conditioning, i.e. incomplete ablation. This is, however, dosage-dependent. Transplantation of the HSCs should then lead to engraftment and repopulation of the immune system, which is demonstrated by the line graphs. Following high-intensity regimens, the cell counts are at near-0 prior to engraftment and the engrafted cells only repopulate the immune system. Following the other regimens, engrafted cells are thought to compete with the remaining immune cells and then out-compete and predominate the old T cell clones. Initially, however, the TCR repertoire is, of course, restricted due to the destruction of T cells, and therefore, T cell diversity is low. Early changes (within 1 year) include the production of de novo immune cells—namely neutrophils, NK, CD8+ T cells and B cells—and/or perhaps repopulation of “old” circulating immune cells, with the later (in around 2–4 years) occurrence of thymic activation or rebound then allowing for the production of a new, more diverse TCR repertoire that is no longer autoreactive or does not allow for the expansion of autoreactive clones

